# Clinical-Pathological profile of head and neck cancers other than squamous cell carcinoma: A retrospective 20-year follow-up study

**DOI:** 10.4317/jced.62968

**Published:** 2025-08-01

**Authors:** Paulo Goberlânio de Barros Silva, Mikaele Zizuino da Silva, Giulianna Aparecida Vieira Barreto, Ana Beatriz Silva Marques Araújo, Thinali Sousa Dantas, Cássia Emanuella Nobrega Malta, Fabrício Bitu Sousa, Marcelo Gurgel Carlos da Silva

**Affiliations:** 1 Hospital Haroldo Juaçaba, Ceará Cancer Institute, Fortaleza, Ceará, Brazil; 2 Christus University Center, Ceara, Brazil

## Abstract

**Background:**

Squamous cell carcinoma (SCC) of the head and neck is the main histological type and the sixth most common cancer in the world. However, tumors other than squamous cell carcinoma can affect the oral cavity, such as salivary gland carcinomas, lymphomas and sarcomas.

**Material and Methods:**

A quantitative, retrospective, observational and cross-sectional study, in which 395 medical records of patients diagnosed/treated with non-SCC head and neck tumors from 2000 to 2020 at the Haroldo Juaçaba Hospital/Ceará Cancer Institute (HHJ/ICC) were analyzed. The data was compared using Pearson’s chi-square test or Fisher’s exact test, Kaplan-Meier overall survival curves were constructed and the Mantel-Cox log-rank test was used.

**Results:**

Salivary gland tumors (1st = Adenoid Cystic Carcinoma), followed by sarcomas (1st = Kaposi’s Sarcoma) and lymphoproliferative tumors (1st = Diffuse Large B-Cell Lymphoma). The majority of the sample were women, with a mean age of 56 and a low level of education. Median overall survival was (95%CI = 57.29-101.71) months, with no difference between the lesion groups (*p*=0.727). Salivary gland tumors and sarcomas affected significantly younger age groups (*p*=0.011). Most of the sample was N0 (*p*=0.006) and multimodal therapy was the preferred choice, especially for salivary gland tumors, sarcomas and melanomas (*p*<0.001). Schooling (*p*=0.007) was inversely associated with overall survival and the independent predictor of death was the presence of lymph nodes (*p*=0.039).

**Conclusions:**

The frequency of non-SCC head and neck tumors is very low. There is no difference between men and women, age is a determining factor in differentiating tumors and lymph node metastasis is the main predictor of survival. In addition, schooling is an important risk factor for mortality in these patients.

** Key words:**Head and Neck Neoplasms, Squamous Cell Carcinoma, Salivary Gland Neoplasms, Survival.

## Introduction

Head and neck cancer is the fifth most common type of cancer in the world. It is more prevalent in males and its occurrence increases with age. The overall survival rate for this cancer is variable, depending on the primary site and the stage of the disease. The development of these head and neck cancers is the result of multifactorial interactions. It is known that tobacco use associated with alcohol consumption is a well-established risk factor for head and neck cancer [1]. According to the National Cancer Institute (INCA) [2], the estimated number of new cases of oral cavity cancer in Brazil for each year of the three-year period from 2023 to 2025 is 15,100 cases.

Squamous cell carcinoma (SCC) of the head and neck is the main histological type, responsible for around 90% of malignancies in these places, and the sixth most common cancer in the world [3]. The profile of head and neck SCCs is predominantly of men aged over 50 or 60 and smokers [4], but since tumors other than squamous cell carcinoma have a very low frequency, the identification of clinical and sociodemographic profiles, as well as prognosis and therapy, is compromised by the low number of epidemiological studies and clinical trials.

Among non-SCC tumors, salivary gland tumors are the most common. Mucoepidermoid carcinoma (MEC) is the most common neoplasm of the salivary glands, accounting for 10 to 15% of all salivary neoplasms and the most common location of CME is the parotid glands [5]. Adenoid cystic carcinoma (ACC) is usually the second most common salivary gland tumor in the head and neck. It can occur in any type of salivary gland, but it most commonly originates in minor salivary glands . It can be classified into cribiform, tubular or solid histological patterns, the latter having the worst prognosis, as it presents cellular pleomorphism and intense mitotic activity [6].

Adenocarcinomas, in turn, encompass a wide variety of histological types of tumors. Those that affect the minor salivary glands are called low-grade polymorphs and are curable in most cases. The polymorphous adenocarcinoma (PAC) has a mean age of diagnosis in the 60s, affecting more women than men in a ratio of 2:1. The palate is the site with the highest incidence of this tumor [7].

Lymphomas account for approximately 5% of all malignant neoplasms in the head and neck region. They are divided into two subgroups, Hodgkin’s Lymphomas (HLs) and Non-Hodgkin’s Lymphomas (NHLs), whose differentiation depends on the presence or absence of Reed-Sternberg cells [8]. In general, HL is more prevalent in patients aged between 20 and 30, while NHL is more frequently diagnosed between 70 and 80 years of age, the latter being the most frequent type of head and neck tumor [8].

The biological behavior and clinical outcome of the different subtypes of the disease are quite variable, which implies different therapeutic modalities. Normally, chemotherapy is the standard therapy of choice for myoloproliferative diseases with possible maintenance treatment in certain subsets of patients [9]. However, the choice depends on the stage of the disease, clinical characteristics including prognostic factors and considerations of potential long-term toxicities of the therapy [10].

Head and neck sarcomas are a rare condition and most arise from soft tissues. They occur more frequently in men at a ratio of 2:1 and the average age of diagnosis is around 50 to 55 years. Therefore, as they are associated with high recurrence and mortality rates, surgical resection with wide margins is the most suitable treatment option. However, the surgical literature still lacks evidence on treatment options for head and neck sarcomas (11).

## Material and Methods

- Study Design

This is a quantitative, retrospective, observational and cross-sectional study, collecting data from patients diagnosed/treated with head and neck tumors other than squamous cell carcinomas, from January 1, 2000 to December 31, 2020, a total of 20 years, at the Haroldo Juaçaba Hospital/Ceará Cancer Institute (HHJ/ICC), a High Complexity Oncology Care Center (CACON) in northeastern Brazil.

This study was based on the design proposed by STROBE-cheklist [12], and was approved by the Ethics Committee of Haroldo Juaçaba Hospital under protocol number 5.100.277. All phases of the study were carried out in accordance with Law 466/12 on research ethics, and the confidentiality of information from the patients’ medical records was guaranteed and kept until the end of the study.

- Search tool and inclusion and exclusion criteria

To select the sample, all head and neck tumors were screened using the Tasy electronic port system at Haroldo Juaçaba Hospital during the period mentioned above. Using the search tool, the ICD10 codes C760 were screened. Category C760 refers to malignant neoplasm of the head, face and neck and is part of the group between C76 and C80 and Chapter II of the ICD 10 book. The code is C760, the description is malignant neoplasm of head, face and neck, classified as no dual classification.

The data was exported to a standard Microsoft Excel spreadsheet containing information such as the medical record number, histopathological diagnosis, sociodemographic data such as age and gender, location of the primary tumor, TNM staging, treatment carried out, date of diagnosis/start of treatment and date of discharge or death to calculate survival

All records with a completed histological diagnosis and at least 75% of the sociodemographic and clinical-pathological data described above were included. In the case of missing data, the medical record number was used to retrieve the electronic or physical medical record and complete the information. Patient records whose physical medical records were needed for complementation but were not available or were ineligible were excluded

- Tumor classification and death data collection

The location of the primary tumor was re-classified as recommended by the WHO’s international classification of diseases ICD-0 into: major salivary glands: parotid gland, submandibular gland, sublingual gland and other major salivary glands, minor salivary glands, lip, tongue, palate, floor of mouth and other parts and unidentified parts of the mouth [13]. The tumor’s pTNM was defined according to the new guidelines proposed by the American Joint Committee on x’Cancer (AJCC) , which determines TNM with T referring to tumor size, N to lymph node involvement and M related to distant metastases [14]. In addition, overall survival was calculated using the difference between the date of surgical removal of the tumor (day, month and year) and the date of death (day, month and year) or the last end of follow-up [4].

For all patients who have not been informed of their death or date of death, their name will be used for obituary tracing in the National Register of Deceased Persons (CNF) (https://falecidosnobrasil.org.br/). The CNF is the largest obituary registry in Brazil and is an advanced tool for searching for deceased people. It is an open-access website which, by searching for an individual’s name, allows you to trace their date of death registered at any registry office in Brazil. CNF Brasil supports relatives and family members of the deceased, preserving their memories by offering a space for tributes and biographies, rescuing the history of each human being.

- Statistical analysis

Data were expressed as absolute and percentage frequencies and compared using Pearson’s chi-square test or Fisher’s exact test. Kaplan-Meier overall survival curves were also constructed and expressed as medians and 95% confidence intervals and compared using the Mantel-Cox log-rank test. In addition, a Cox regression model (multivariate analysis) was used with the significant variables to analyze the predictors of prognosis.

## Results

Between 2000 and 2020, 395 non-squamous cell carcinoma tumors were diagnosed at the Haroldo Juaçaba Hospital / Cancer Institute of Ceará, and it can be seen that the most prevalent tumors are salivary gland tumors with 302 (76.5%) cases, followed by myeloproliferative disorders with 50 (12.6%) cases. In addition, there were only 9 (2.3%) cases of bone or soft tissue sarcomas and 9 (2.3%) cases of head and neck melanomas. Other disorders, such as large cell carcinoma, accounted for 25 (6.3%) cases.

Of the salivary gland tumors, Adenoid Cystic Carcinoma (*n*=94; 31%) was the most frequent tumor, followed by Adenocarcinoma (*n*=74; 24.8%) and Mucoepidermoid Carcinoma (*n*=67; 22.1%), respectively. The other tumors are described in  Table 1. With regard to myeloproliferative disorders, diffuse large B-cell lymphoma (*n*=11; 22%) was the most common. When bone and connective tissue sarcomas and melanomas were analyzed, the most frequent were Kaposi’s sarcoma (*n*=4; 44.4%) and malignant melanoma (*n*=8; 88.9%), respectively.

Of the 395 patients evaluated, most of the sample was made up of females (*n*=206; 52.2%) with a mean age of 56 years (56.3±18.7, 9-99), with the youngest patient diagnosed at 9 years and the oldest at 99 years of age. The age group showed an increasing incidence in each decade of life, with those over 70 having the highest prevalence (*n*=98; 24.8%). With regard to race, brown people (*n*= 267; 67.6%) were the most frequent and with regard to schooling, this frequency was more significant in patients with incomplete primary education (*n*=73; 27.3%) and complete primary education (*n*=71; 26.6%).

With regard to origin, patients from the capital (*n*=199; 50.4%), followed by those from the countryside (*n*=136; 34.4%) were the most affected by non-squamous cell carcinoma mouth tumors. As for staging, this was described in only 176 cases, with T2 tumors (*n*=52; 29.5%) being the most frequent, followed by T1 (*n*=47; 26.7%). Lymph node metastasis was described in 157 cases, with the absence of lymph node metastasis (*n*=119; 75.8%) being the most frequent. The presence or absence of distant metastasis was described in only 146 cases, with only 5 patients diagnosed with distant metastasis (*n*=5; 3.4%). Of the treatments carried out, most patients were treated with radiotherapy (*n*=95; 24.1%), followed by surgery + RT (*n*=93; 23.5%). The other treatments are shown in  Table 2.

After associating the variables with the histological type, it was possible to observe that gender (*p*=0.943), race (*p*=0.160), schooling (*p*=0.328), origin (*p*=0.368), T (*p*=0.310) and M (*p*=0.496) staging showed no significant association with the histological type. However, with regard to age, it can be seen that salivary gland tumors and sarcomas were significantly more frequent in patients up to 50 years old, while the other types of tumors were significantly more frequent in patients over 50 (*p*=0.011). When analyzing lymph node metastasis, it was possible to observe that patients with salivary gland tumors, sarcomas, melanomas, myeloproliferative disorders were predominantly N0, while the other tumors other than squamous cell carcinoma were N+ (*p*=0.006). In addition, with regard to treatment, both salivary gland tumors and sarcomas were mostly treated by RT or surgery + RT, while melanomas were significantly treated by surgery + RT or RT + QT. Myeloproliferative disorders were treated more significantly by chemotherapy and the other tumors showed a significant amount of treatment abstention (*p*<0.001).

**Figure 1:**
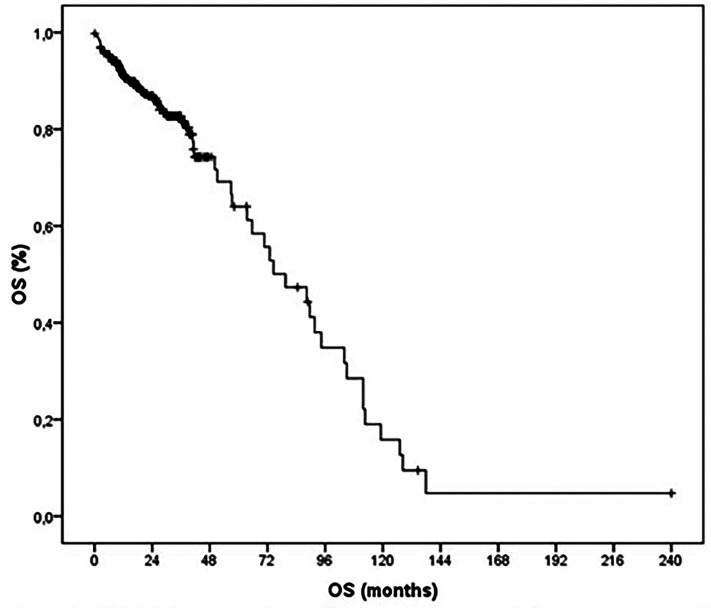
Kaplan Meier curve of overall survival of patients with mouth cancers other than suamous cell carcinoma by histological type at the Haroldo Juaçaba Hospital/Cancer Institute of Ceará, 2000 to 2020.

Figure 1 shows the Kaplan Meier overall survival curve for non-Squamous Cell Carcinoma tumors diagnosed at the Haroldo Juaçaba Hospital / Instituto do Câncer do Ceará with a maximum follow up of up to 20 years. Of the 395 patients, the median overall survival was 59.50 months, with 79.5% of patients alive during this median time.

In  Table 3, gender (*p*=0.363), age (*p*=0.096), race (*p*=0.557), origin (*p*=0.080), T (*p*=0.442), M (*p*=0.469) and histological type (*p*=0.727) did not influence overall survival. However, it can be seen that schooling (*p*=0.007) was directly associated with life expectancy, i.e. the lower the schooling, the lower the survival of patients with mouth cancers other than squamous cell carcinoma. With regard to treatment, it was observed that patients treated with radiotherapy, chemotherapy or surgery + RT had the highest overall survival rates (*p*=0.001). In addition, patients with N2 and N3 lymph node metastases had lower survival rates than N0 and N1 (*p*=0.006).

Finally, the multivariate analysis showed that the main predictor of death was the presence of lymph node metastasis (*p*=0.039), with a mortality risk 1.87 times (CI95% = 1.03-3.38) higher when compared to non-SCC tumors without lymph node metastasis, ( Table 4).

## Discussion

In our study, the most frequent salivary gland tumor was Adenoid Cystic Carcinoma, followed by Adenocarcinoma and Mucoepidermoid Carcinoma. Histopathological records including 574 patients (23%) diagnosed with malignant salivary gland tumors were analyzed, similarly identifying a higher frequency of mucoepidermoid carcinomas (27.9%), adenoid cystic carcinomas (20.9%), acinic cell carcinomas (14.6%) and polymorphous adenocarcinomas (2.8%) according to an 11-year retrospective study [15]. Despite the variation in distribution among salivary gland tumors, adenoid cystic carcinoma, adenocarcinoma and mucoepidermoid carcinoma are the most prevalent, since this variation in classification can be influenced by numerous factors, such as the site affected and the population studied.

With regard to head and neck sarcomas, they account for 5 to 10% of all sarcomas and when compared to head and neck tumors, they comprise a small proportion, which is estimated at 1% [16]. In our study, when analyzing 395 patients, the proportion of sarcomas was only 2.3%, highlighting the rarity of these tumors.

Malignant melanomas occurring in the head and neck region account for around a fifth of all melanomas, with the most affected sites being the face, scalp and neck, outer ear and eyelid [17]. In our study, the occurrence of melanomas was lower, at only 2.3%. Other studies show that the head and neck are rarely affected, but exclusive studies of melanoma highlight the head and neck as the second or third topographical site for melanoma [18].

According to the estimated rates, based on the Global Cancer Observatory database presented in Wang’s study published in 2023 [19], lymphomas are classified as the fifth to ninth most common cancer in most countries. In our study, myeloproliferative disorders accounted for 12.6% of cases, with diffuse large B-cell lymphoma being the most common neoplasm, followed by malignant non-Hodgkin’s lymphoma. The study published by Ansell in 2015 (9) describes non-Hodgkin’s lymphoma as a malignant lymphoid neoplasm with multiple subtypes, with diffuse large B-cell lymphoma being the most common histological subtype, affecting one third of all non-Hodgkin’s lymphomas, which corroborates the findings in our study.

With regard to gender, the majority of patients were female and of these, the highest prevalence was of patients affected by salivary gland tumors. A retrospective study of 195 cases of CAC was carried out and identified a similar clinical-pathological profile with a higher prevalence in females between the fifth and sixth decade of life [20]. Filho *et al*. [21] 9 analyzed 193 patients with salivary gland tumors and also described that the majority of patients were women (55.1%) diagnosed after the age of 50, corroborating our data. As squamous cell carcinoma (91.4%), which is the main histological type diagnosed in the head and neck, mainly affects males aged between 61 and 70 [4], the exclusion of these tumors and the higher frequency of salivary gland tumors in our sample lowers the average age and biases the diagnosis towards women [22].

Head and neck sarcomas also have a higher proportion of men than women [16], similar to our study, but as their proportion is lower, women are still the majority in our sample. The average age at diagnosis of our patients was also lower, although the average age of these head and neck tumors is 58 [16].

In the analysis of myeloproliferative disorders, which include the occurrence of non-Hodgkin’s lymphoma and Burkitt’s lymphoma, in our study there was no great divergence in relation to the sexes, a result that diverges from the findings by Chiu and Hou [23] in which the general rate of NHL is around 50% higher in men than in women, but corroborates the findings of average age at diagnosis observed in our study [19].

The association between schooling and the prognosis of these tumors is interesting to describe. Patients with a low level of education (56.9%) were the majority in our study and had a higher mortality rate. In a European multicenter study, no significant association was observed with the level of education, but specifically studying diffuse large B-cell lymphomas, a significantly lower risk was observed for individuals with a higher level of education [24]. In tumors with great social appeal in which the risk factors can be controlled, such as SCC, this is expected [4], but in tumors with rare involvement it is something that needs to be pointed out, since in Brazil diagnosis tends to be later the lower the level of education, directly influencing survival. In studies evaluating melanomas, for example, this association has also been observed [25].

However, in a multivariate analysis, lymph node involvement was the main predictor of mortality. Tsai *et al*. [26] described that the presence of cervical lymph node metastasis in patients with head and neck SCC reduces the overall survival of these patients by around 50% when compared to patients without lymph node metastasis. However, in our study with non-SCC tumors, we found that this parameter was also very important as a factor influencing survival. In malignant salivary gland tumors, this factor did not significantly influence mortality or survival time when many salivary gland tumors are analyzed together [21], but an increase in mortality due to the presence of lymph node invasion was also observed when evaluating mucoepidermoid carcinomas [27].

It is important to highlight the limitation of our study, as it is a retrospective study, the absence of more than half of the TNM staging of the patients in the sample, since this is known to be an extremely important variable for classifying and comparing patients with different types of cancer, facilitating the analysis of clinical data, prognosis and treatment results. As such, the study found it difficult to relate this variable and showed no statistical difference, even though lymph node metastasis was the most important factor.

However, this is the first study to carry out a 20-year survey of non-SCC head and neck tumors. Thus, although SCC tumors are mostly 90 to 95% of head and neck tumors, knowing about non-SCC tumors is crucial for local and regional health authorities to be able to prepare health services to diagnose these types of tumors.

## Conclusions

The frequency of non-SCC head and neck tumors is very low. There is no difference between men and women, age is a determining factor in differentiating tumors and lymph node metastasis is the main predictor of survival. In addition, schooling is an important risk factor for mortality in these patients and median survival is relatively higher than expected.

## Figures and Tables

**Table 1:** Distribution of mouth cancers other than squamous cell carcinoma at the Haroldo Juaçaba Hospital / Ceará Cancer Institute, 2000 to 2020.

	n	%
Salivary gland tumors	302	76,5
Adenoid cystic carcinoma	94	31,0
Adenocarcinoma	74	24,8
Mucoepidermoid carcinoma	67	22,1
Acinar cell carcinoma	12	4,0
Epithelial-myoepithelial carcinoma	8	2,6
Pleomorphic carcinoma	8	2,6
Myoepithelial carcinoma	7	2,3
Mucinous adenocarcinoma	6	2,0
Papillary adenocarcinoma	6	2,0
Clear cell adenocarcinoma	5	1,7
Infiltrating ductal carcinoma	5	1,7
Basal cell adenocarcinoma	3	1,0
Carcinoma ex pleomorphic adenoma	3	1,0
Adenosquamous carcinoma	2	0,7
Cribiform carcinoma in situ	1	0,3
Adenoid squamous cell carcinoma	1	0,3
Sarcomas of bone and connective tissue	9	2,3
Kaposi's sarcoma	4	44,4
Botryoid sarcoma	3	33,3
Rhabdomyosarcoma	1	11,1
Undifferentiated sarcoma	1	11,1
Melanoma	9	2,3
Malignant melanoma	8	88,9
Lentiginous melanoma of the mucosa	1	11,1
Myeloproliferative	50	12,6
Diffuse large B-cell lymphoma	11	22,0
Malignant non-Hodgkin's lymphoma	9	18,0
Diffuse immunoblastic malignant large B-cell lymphoma	8	16,0
B-cell marginal zone lymphoma	5	10,0
Mature T-cell lymphoma	3	6,0
Lymphoepithelioma-like carcinoma	2	4,0
Mantle cell lymphoma	2	4,0
Follicular lymphoma	2	4,0
Malignant small B-cell lymphoma	2	4,0
Plasmacytoma	2	4,0
Burkitt's lymphoma	1	2,0
Hodgkin's lymphoma	1	2,0
Nodular sclerosing Hodgkin's lymphoma	1	2,0
Malignant lymphoma	1	2,0
Other	25	6,3
Large cell carcinoma	16	64,0
Sarcomatoid carcinoma	4	16,0
Carcinosarcoma	2	8,0
Spindle cell squamous cell carcinoma	1	4,0
Neuroendocrine carcinoma	1	4,0
Undifferentiated tumor	1	4,0

Data expressed as absolute frequency and percentage.

**Table 2:** Sociodemographic and clinical characterization of mouth cancers other than squamous cell carcinoma by histological type at the Haroldo Juaçaba Hospital / Cancer Institute of Ceará, 2000 to 2020.

		Histological type					p- Value
	Total	T. Gl. Saliv.	Sarcomas	Melanomas	Myeloproliferative	Others	
Sex							
Male	189(47.8%)	142(47.0%)	5(55.6%)	5(55.6%)	24(48.0%)	13(52.0%)	0,953
Female	206(52.2%)	160(53.0%)	4(44.4%)	4(44.4%)	26(52.0%)	12(48.0%)	
Age (56.3±18.7, 9-99)							
Up to 30	37(9.4%)	35(11.6%)*	2(22.2%)*	0(0.0%)	0(0.0%)	0(0.0%)	0,011
31-40	54(13.7%)	41(13.6%)	2(22.2%)*	3(33.3%)	6(12.0%)	2(8.0%)	
41-50	55(13.9%)	48(15.9%)	2(22.2%)*	1(11.1%)	3(6.0%)	1(4.0%)	
51-60	68(17.2%)	48(15.9%)	1(11.1%)	1(11.1%)	10(20.0%)	8(32.0%)*	
61-70	83(21.0%)	65(21.5%)	1(11.1%)	2(22.2%)	13(26.0%)	2(8.0%)	
>70	98(24.8%)	65(21.5%)	1(11.1%)	2(22.2%)	18(36.0%)	12(48.0%)*	
Race							
White	128(32.4%)	103(34.1%)	0(0.0%)	1(11.1%)	16(32.0%)	8(32.0%)	0,160
Brown	267(67.6%)	199(65.9%)	9(100.0%)	8(88.9%)	34(68.0%)	17(68.0%)	
Education							
None	36(13.5%)	25(12.1%)	0(0.0%)	3(60.0%)	5(15.6%)	3(15.8%)	0,328
Fund incomplete	73(27.3%)	55(26.6%)	2(50.0%)	2(40.0%)	7(21.9%)	7(36.8%)	
Fund completo	71(26.6%)	54(26.1%)	2(50.0%)	0(0.0%)	10(31.3%)	5(26.3%)	
Medium	55(20.6%)	46(22.2%)	0(0.0%)	0(0.0%)	6(18.8%)	3(15.8%)	
Complete university degree	32(12.0%)	27(13.0%)	0(0.0%)	0(0.0%)	4(12.5%)	1(5.3%)	
Origin							
Capital	199(50.4%)	155(51.3%)	2(22.2%)	3(33.3%)	26(52.0%)	13(52.0%)	0,368
Metropolitan area	60(15.2%)	45(14.9%)	3(33.3%)	1(11.1%)	5(10.0%)	6(24.0%)	
Inside	136(34.4%)	102(33.8%)	4(44.4%)	5(55.6%)	19(38.0%)	6(24.0%)	
T							
1	47(26.7%)	39(25.7%)	0(0.0%)	0(0.0%)	4(36.4%)	4(40.0%)	0,310
2	52(29.5%)	50(32.9%)	0(0.0%)	1(50.0%)	1(9.1%)	0(0.0%)	
3	39(22.2%)	33(21.7%)	1(100.0%)	0(0.0%)	3(27.3%)	2(20.0%)	
4	38(21.6%)	30(19.7%)	0(0.0%)	1(50.0%)	3(27.3%)	4(40.0%)	
N							
0	119(75.8%)	107(78.7%)*	1(100.0%)*	1(100.0%)*	6(66.7%)*	4(40.0%)	0,006
1	11(7.0%)	11(8.1%)	0(0.0%)	0(0.0%)	0(0.0%)	0(0.0%)	
2	16(10.2%)	13(9.6%)	0(0.0%)	0(0.0%)	0(0.0%)	3(30.0%)*	
3	11(7.0%)	5(3.7%)	0(0.0%)	0(0.0%)	3(33.3%)	3(30.0%)*	
M							
0	141(96.6%)	122(97.6%)	1(100.0%)	1(100.0%)	8(88.9%)	9(90.0%)	0,496
1	5(3.4%)	3(2.4%)	0(0.0%)	0(0.0%)	1(11.1%)	1(10.0%)	
Treatment							
None	63(15.9%)	43(14.2%)	0(0.0%)	2(22.2%)*	13(26.0%)*	5(20.0%)*	<0,001
Surgery	65(16.5%)	52(17.2%)	2(22.2%)	2(22.2%)	5(10.0%)	4(16.0%)	
RT	95(24.1%)	80(26.5%)*	2(22.2%)*	0(0.0%)	7(14.0%)	6(24.0%)	
QT	32(8.1%)	13(4.3%)	2(22.2%)	1(11.1%)	16(32.0%)*	0(0.0%)	
Surgery + RT	93(23.5%)	81(26.8%)*	2(22.2%)*	2(22.2%)*	4(8.0%)	4(16.0%)	
RT + QT	34(8.6%)	22(7.3%)	1(11.1%)	2(22.2%)*	4(8.0%)	5(20.0%)*	
Surgery + RT + QT	13(3.3%)	11(3.6%)	0(0.0%)	0(0.0%)	1(2.0%)	1(4.0%)	

*p <0.05, Perason’s chi-square test or Fisher’s exact test (n, %).

**Table 3:** Survival analysis of patients with non-squamous cell mouth cancers by histological type at the Haroldo Juaçaba Hospital / Ceará Cancer Institute, 2000 to 2020.

	Total	OS	Median (CI95%)	p-Value
Total	395	315(79.7%)	79.50(57.29-101.71)	-
Sex				
Male	189	140(74.1%)	89.60(68.88-110.32)	0,363
Female	206	175(85.0%)	57.20(38.33-76.07)	
Age				
Up to 30	37	33(89.2%)	105.00(92.14-117.57)	0,096
31-40	54	45(83.3%)	57.20(44.56-69.84)	
41-50	55	47(85.5%)	79.50(54.31-104.69)	
51-60	68	57(83.8%)	103.90(45.76-162.04)	
61-70	83	63(75.9%)	65.60(45.81-85.39)	
>70	98	70(71.4%)	74.50(33.55-115.45)	
Race				
White	128	98(76.6%)	103.90(29.42-178.38)	0,557
Brown	267	217(81.3%)	74.50(56.84-92.16)	
Education				
Illiterate	36	19(52.8%)	65.60(14.47-116.73)	0,007
Elementary school incomplete	73	47(64.4%)	70.70(29.07-112.33)	
Complete primary education	71	55(77.5%)	94.40 (35.17-121.15)	
Medium	55	48(87.3%)	57.20(5.32-109.08)	
Complete university degree	32	32(100.0%)	NC	
Origin				
Capital	199	167(83.9%)	103.90(80.38-127.42)	0,080
Metropolitan area	60	43(71.7%)	56.80(32.01-81.59)	
Inside	136	105(77.2%)	70.70(53.34-88.06)	
T				
1	47	40(85.1%)	70.70(6.20-135.20)	0,442
2	52	42(80.8%)	88.30(30.18-146.42)	
3	39	32(82.1%)	89.60(0.00-190.81)	
4	38	25(65.8%)	65.60(36.63-94.57)	
N				
0	119	98(82.4%)	74.50(48.98-100.02)	0,006
1	11	11(100.0%)	NC	
2	16	9(56.3%)	30.00(1.70-58.30)	
3	11	8(72.7%)	50.00(3.41-57.12)	
M				
0	141	115(81.6%)	88.30(49.57-127.03)	0,469
1	5	3(60.0%)	65.60(0.00-146.85)	
Treatment				
None	63	50(79.4%)	72.90(61.70-84.10)	0,001
Surgery	65	55(84.6%)	63.50(58.12-77.10)	
RT	95	79(83.2%)	103.90(82.07-125.73)	
QT	32	23(71.9%)	119.10(91.12-132.14)	
Surgery + RT	93	77(82.8%)	89.60(43.86-135.34)	
RT + QT	34	24(70.6%)	50.00(23.14-73.15)	
Surgery + RT + QT	13	7(53.8%)	30.00(5.65-54.35)	
Histological type				
Salivary gland tumors	302	242(80.1%)	89.60(68.86-110.34)	0,727
Sarcomas	9	8(88.9%)	36.30(30.10-39.15)	
Melanomas	9	4(44.4%)	41.20(40.57-41.83)	
Myeloproliferative	50	41(82.0%)	50.00(32.32-67.68)	
Others	25	20(80.0%)	74.50(68.10-82.10)	

*p <0.05, Log-Rank Mantel-Cox test; NC = Not calculated; OS = overall survival; CI95% = 95% confidence interval.

**Table 4:** Multivariate analysis of factors influencing survival in patients with non-squamous cell mouth cancers by histological type at the Haroldo Juaçaba Hospital / Ceará Cancer Institute, 2000 to 2020.

	p-Value	HR (CI95%)
Risk of death		
Sex	0,219	2,20 (0,62-7,77)
Age	0,168	1,19 (0,93-1,53)
Race	0,931	1,05 (0,37-2,92)
Education	0,806	1,06 (0,66-1,70)
Origin	0,069	1,74 (0,96-3,18)
T	0,510	1,20 (0,69-2,09)
N	*0,039	1,87 (1,03-3,38)
M	0,078	5,62 (0,82-38,39)
Treatment	0,054	1,01 (0,99-1,02)
Histological type	0,243	0,79 (0,52-1,18)

*p <0.05, Cox regression; HR = Hazard risk; CI95% = 95% confidence interval.

## Data Availability

N/A.
